# Performance of empirical and model-based classifiers for detecting sucrase-isomaltase inhibition using the ^13^C-sucrose breath test

**DOI:** 10.1088/1752-7163/ad748d

**Published:** 2024-09-10

**Authors:** Hannah Van Wyk, Gwenyth O Lee, Robert J Schillinger, Christine A Edwards, Douglas J Morrison, Andrew F Brouwer

**Affiliations:** 1 Department of Epidemiology, University of Michigan, 1415 Washington Heights, Ann Arbor, MI 48109, United States of America; 2 Rutgers Global Health Institute, 112 Paterson St., New Brunswick, NJ 08901, United States of America; 3 Scottish Universities Environmental Research Centre (SUERC), University of Glasgow, Rankine Avenue, East Kilbride G750QF, United Kingdom; 4 School of Medicine, Dentistry and Nursing, University of Glasgow, New Lister Building, Alexandra Parade, Glasgow G31 2ER, United Kingdom

**Keywords:** environmental enteric dysfunction, ^13^C-sucrose breath test, sucrase-isomaltase inhibition, mechanistic model, classifier

## Abstract

The ^13^C-sucrose breath test (^13^C-SBT) has been proposed to estimate sucrase-isomaltase (SIM) activity and is a promising test for SIM deficiency, which can cause gastrointestinal symptoms, and for intestinal mucosal damage caused by gut dysfunction or chemotherapy. We previously showed how various summary measures of the ^13^C-SBT breath curve reflect SIM inhibition. However, it is uncertain how the performance of these classifiers is affected by test duration. We leveraged ^13^C-SBT data from a cross-over study in 16 adults who received 0, 100, and 750 mg of Reducose, an SIM inhibitor. We evaluated the performance of a pharmacokinetic-model-based classifier, $\rho $, and three empirical classifiers (cumulative percent dose recovered at 90 min (cPDR90), time to 50% dose recovered, and time to peak dose recovery rate), as a function of test duration using receiver operating characteristic (ROC) curves. We also assessed the sensitivity, specificity, and accuracy of consensus classifiers. Test durations of less than 2 h generally failed to accurately predict later breath curve dynamics. The cPDR90 classifier had the highest ROC area-under-the-curve and, by design, was robust to shorter test durations. For detecting mild SIM inhibition, $\rho $ had a higher sensitivity. We recommend ^13^C-SBT tests run for at least a 2 h duration. Although cPDR90 was the classifier with highest accuracy and robustness to test duration in this application, concerns remain about its sensitivity to misspecification of the CO_2_ production rate. More research is needed to assess these classifiers in target populations.

## 1. Introduction

Sucrase-isomaltase (SIM) is an intestinal glucosidase enzyme that catalyzes the hydrolysis of carbohydrates [1]. Deficiency of SIM can be genetic, as in the case of congenital SIM deficiency (CSID), which results in gastrointestinal symptoms from the fermentation of undigested carbohydrates by microbes in the colon [2]. SIM is produced at villous tips, and so deficiency of SIM can also be a downstream effect of damage to the intestinal mucosa, such as that caused by gut dysfunction or cytotoxic chemotherapy [3, 4]. Of particular interest to the authors is environmental enteric dysfunction (EED), a gut dysfunction characterized by atrophy of the small intestinal villi, resulting in increased intestinal permeability and nutrient malabsorption. It is thought to be highly prevalent among people in low- and middle-income countries who lack access to improved water, sanitation, and hygiene [5] and are therefore repeatedly exposed to enteric pathogens [6, 7]. The downstream impacts of EED include stunting in infants and young children [8], which impacts about 150 million children globally.

The standard method of detecting intestinal mucosal damage is by the identification of histological features in small intestinal biopsies [4, 9, 10]. However, biopsies are invasive and expensive, limiting the ability to accurately, efficiently, and inexpensively identify EED and other forms of gut dysfunction, especially in low-resource settings [11]. The ^13^C sucrose breath test (^13^C-SBT) and related ^13^C substrate breath tests have been proposed as non-invasive alternatives to biopsies and have been used to investigate EED [12–14], celiac disease [15], CSID [16–18], functional bowel disorders [19], and mucositis induced by cytotoxic chemotherapy [4, 20]. The ^13^C-SBT is a stable-isotope breath test in which an individual ingests a dose of non-radioactive, ^13^C-labeled substrate, which is digested, absorbed, and metabolized, appearing on the breath as ^13^CO_2_. Slower recovery of the tracer on the breath indicates reduced SIM activity.

Although the ^13^C-SBT is attractive as a potential, non-invasive test of EED and other causes of SIM deficiency, it also has some limitations, which are common across ^13^C breath tests. Traditional measures used to interpret breath tests consist of empirically fitting a parametric curve to the percent dose recovery rate (PDRr) and calculating summary statistics, such as the cumulative percent dose recovered at 90 min (cPDR90), the time to peak PDRr (${T_{{\text{peak}}}}$), or the time to 50% dose recovered (${T_{50}}$) [21, 22]. However, empirical measurements do not necessarily capture the underlying biological processes giving rise to the PDRr curve, and thus any diagnosis based on these measures may be confounded by multiple aspects of the metabolism, some of which are unrelated to gut function. To address this concern we developed a mechanistic, pharmacokinetic model whose parameters represent the underlying biological processes occurring in the metabolism of the ^13^C-labeled sucrose tracer [23]. A model-based diagnostic *ρ* performed comparably to the highest-performing summary statistics in identifying experimentally induced SIM inhibition in healthy adults [24].

In this analysis, we revisit these exploratory experiments to assess how the performance of the four highest performing classifiers, namely *ρ*, cPDR90, ${T_{{\text{peak}}}}$ and ${T_{50}}$, depend on the test duration. While experiments establishing and evaluating the ^13^C-SBT have used test durations of 4–8 h [13, 23], there is a strong need to reduce the testing burden on participants, particularly for the target population of infants and children under 5 years. Additionally, because cPDR90, ${T_{{\text{peak}}}}$, ${T_{50}},\,$and *ρ* capture different information about the breath curve, we will determine if consensus classifiers combining two or more classifiers can produce a more reliable diagnosis. In this research, we address these research gaps by assessing the accuracy of ^13^C-SBT curve projections based on shorter test duration, the performance of these four classifiers across test durations, and performance of consensus classifiers.

## 2. Methods

### 2.1. Data

The ^13^C-SBT breath curves used in this study were obtained in a crossover study conducted in Glasgow, United Kingdom, as previously described [24]. In brief, eighteen healthy adults were recruited to complete three breath test experiments under different experimental conditions designed to simulate different degrees of SIM inhibition. In this analysis, we only use data from the 16 participants who completed all three breath tests. The participants consisted of 8 female and 8 male participants with a mean age of 24.2 (SD = 5.0) and mean BMI of 24.5 (SD = 5.2). Participants were instructed to follow a low ^13^C diet for the 3 d preceding the experiments and to fast for 8 h prior to the test. A low ^13^C diet avoids plants that photosynthesize using the C4 pathway (e.g. corn or cane sugar) or the meat of animals predominantly fed C4 plants (e.g. chicken) [25]. Deviation from a low ^13^C diet can cause the baseline ^13^CO_2_ production to not be stable over the test period, although this concern is greatly reduced when using a highly enriched tracer rather than a naturally enriched tracer.

In the first experiment, participants ingested 25 mg (0.84 mmol ^13^C) of highly enriched sucrose (⩾99 atom% enriched; Sigma-Aldrich) to complete a baseline test. Breath samples were collected every 15 min for 4 h into 12 ml Exetainer breath-sample vials (Labco, United Kingdom). The relative difference in parts per thousand between the ratio ${R_{\text{s}}} = $[^13^C]/[^12^C] in the sample and the (${R_{{\text{std}}}}$) of the laboratory CO_2_ standard (calibrated to the international Vienna Peedee Belemnite calibration standard, *R* = 0.011 2372) were determined by isotope ratio mass spectrometry (IRMS, AP-2003, Manchester, United Kingdom). Details on how this measurement was converted to percent dose recovery rates are described in previous work [23]; these calculations include an estimate of carbon dioxide production, V_CO2_, which is estimated based on body size using a standard formula [26]. In the remaining experiments, participants were given 100 and 750 mg (in random order) of Reducose® (Phynova Group Ltd, Oxford, UK), a mulberry leaf extract (MLE) containing 5% 1-Deoxynojirimycin (an active *α*-glucosidase inhibitor) immediately prior to ingesting the 25 mg sucrose. MLE has been shown to function as an intestinal SIM inhibitor, thus it is expected to induce similar ^13^CO_2_ excretion patterns to those that would be observed in patients with EED or other conditions resulting in a loss of SIM activity. The low dose of 100 mg MLE was given to induce mild SIM inhibition, and the high dose of 750 mg was given to induce severe inhibition. In some cases, participants with the high dose of MLE exhibited spikes in their PDRr breath curve caused by unmetabolized sucrose tracer entering the colon and being metabolized by microbes; these parts of the curve are excluded from analysis as they do not represent the participant’s metabolism. Investigators received written informed consent from all participants and the study design was approved by the University of Glasgow College of Medical Veterinary and Life Sciences Research Ethics Committee (Application Number: 200190155). This study was performed in accordance with the Declaration of Helsinki. The data underlying are available at [27].

### 2.2. Mechanistic model

In previous work [23], we developed a mechanistic, compartmental differential equation model that captured ^13^C-SBT breath curve dynamics and was practically identifiable, i.e. had parameters that could be uniquely estimated from data. In this model, the breath curve dynamics can be approximated as a combination of a gamma-distributed process with pharmacokinetic rate parameter *ρ*/2 and shape parameter 2 and an exponentially distributed process with rate parameter *πρ*. Because of the limitations of only observing the breath, the specific metabolic processes that these model processes represent are unknown *a priori*. In other previous work, we demonstrated that both SIM inhibition and the difference between fructose and glucose in the transport to and metabolism by the liver were reflected in the gamma-distributed process [24]. In the model, we also account for the fraction of ^13^C that is exhaled, *κ*, as opposed to being secreted in urine or sequestered in adipose tissue.

When *π* ≠ 1, the closed-form solution for PDRr is

\begin{equation*} {y\left( t \right) = \frac{{100\kappa \pi \rho }}{{{{\left( {1 - \pi } \right)}^2}}}\left( {{e^{ - \pi \rho t}} + \left( {\left( {\pi - 1} \right)\rho t - 1} \right){e^{ - \rho t}}} \right),\,}\end{equation*}

and the cPDR is given by

\begin{align*} {Y\left( t \right) = \,100\kappa \left( {1 - \frac{{{e^{ - \pi \rho t}}\, + \left( {\left( {\pi - 1} \right)\rho t + \pi - 2} \right)\pi {e^{ - \rho t}}\,}}{{{{\left( {1 - \pi } \right)}^2}}}} \right),} \end{align*}

which is the area under the curve (integral) of the PDRr curve from time 0 to time *t*. The classifiers we consider in this analysis are all obtained directly from the above equations: cPDR90 = *Y*(90), ${T_{{\text{peak}}}} = \mathop {{\text{argmax}}}\limits_t y\left( t \right)$, ${T_{50}}\left( \omega \right) = \{ t\,|Y\left( t \right) = \frac{{Y\left( \omega \right)}}{2}\} $, where $\omega \,$is the test length, and $\rho $ is the model-based classifier based on previous work [23]. Note that the definition of ${T_{50}}$ used here, 50% of the cumulative percent dose recovered at test length $\omega $, is different from previous work [23], which defined it as time to recovery of 50% of the dose given. We use our definition here because most test participants do not recover 50% of the full dose over the testing period, especially in the case of mild-to-severe SIM inhibition.

### 2.3. Parameter estimation

We estimated the parameter set $\theta = \left\{ {\rho ,\pi \rho ,\kappa } \right\}$ corresponding to the best fit model by minimizing the negative log-likelihood (NLL), given by

\begin{align*} \textrm{NLL}\left( \theta \right)&amp; = \frac{n}{2}\textrm{log}(2\overline{\pi}) + \frac{n}{2}{\textrm{log}}\left( {{\sigma ^2}} \right)\nonumber\\ &amp; \quad + \frac{1}{{2{\sigma ^2}}}\sum_i {{\left( {y\left( {\theta ; {t_i}} \right) - {z_i}} \right)}^2} \end{align*}

where $y\left( {\theta ;\,{t_i}} \right)$ is the value of the modeled PDR at time ${t_i}$, $n$ is the number of data points, $\overline{\pi} $ is the mathematical circle constant, $\sigma $ is the standard deviation previously estimated to be 0.555 from best-fit curves [23], and ${t_i}$ is the time at which measurement ${z_i}$ was taken. In the case where the peak PDRr is not observed during the testing period, which was common among the 750 mg MLE samples, ${T_{{\text{peak}}}}$and $\kappa $ are not identifiable. In this case, we added a penalty of size $0.1\kappa $ onto the NLL to force the optimizer to select lower values of $\rho $. This forces the optimizer to choose larger values of $\pi \rho $ that generate more realistic PDRr curves that do not extend over unrealistically long periods of time.

### 2.4. Analytic approach

The three objectives of this analysis were to (1) compare the accuracy of model projections as a function of test duration, (2) compare the performance of cPDR, *T*_peak_, ${T_{50}}$, and *ρ*, as a function of test duration, and (3) assess the performance of consensus classifiers that combine two or more of the single classifiers. In this analysis, we examined test durations of 60, 90, 120, and 240 min. The following analysis plan outlines our approach:

(1) *Comparing model fits for 60, 90, 120, and 240 min duration tests.* For each participant *j*, we estimated ${\hat \theta _{60,j}},{\hat \theta _{90,j}},{\hat \theta _{120,j}}$, and ${\hat \theta _{240,j}}$, corresponding to the nine parameters that minimized the NLL for the baseline, 100 mg MLE, and 750 mg MLE breath curves, assuming that we only had the data from the first 60, 90, 120, and full 240 min, respectively. Then, to compare the model fits for the 60, 90, and 120 min tests to the full dataset, we simulated the model for 240 min using each parameter set and calculated the NLL from each simulation against the full 240 min data.
(2) *Comparing receiver operator characteristic (ROC) curves for ρ, cPDR,* ${T_{50}}$ *and* ${T_{{\text{peak}}}}$ *for 60, 90, 120, and 240 min duration tests.* We first noted that breath test curves that are initially slower (have a lower PDRr) typically also sustain a higher PDRr longer than the faster curves, allowing them to ‘catch up’ to cumulative dose recovered of faster curves over time. Therefore, the value of cPDR at a later time may be a less effective classifier than the value at an earlier time, and the optimal cPDR should be near the median *T*_peak_. Thus, we first determined which cPDR classifier (cPDR60, cPDR90, cPDR120, or cPDR240) resulted in the most accurate classification using ${\hat \theta _{240,j}}$. As discussed in the results, we selected cPDR90. Then, we simulated the model for each parameter set *θ_60,j_, θ_90,j_*, ${\theta _{120,j}}$, and ${\theta _{240,j}}$, and estimated *ρ*, cPDR90, ${T_{50}}$ and ${T_{{\text{peak}}}}$ in each case. We generated ROC curves (which plot the true positive rate against the false positive rate as the classification threshold is varied) for all 12 combinations of test duration and classifier, for each of 4 groupings of the MLE experiments, corresponding to different clinical scenarios:
  1. Detection of *any* SIM inhibition (baseline versus *either* 100 or 750 mg MLE),
  2. Distinguishing between severe SIM inhibition vs none-to-mild (baseline or 100 mg MLE versus 750 mg MLE),
  3. Detection of mild SIM inhibition (i.e. baseline versus 100 mg MLE),
  4. Detection of severe SIM inhibition (baseline versus 750 mg MLE).
  The goal of the first two diagnostic groupings is to offer a single metric that captures the test’s ability to generate a binary diagnosis of SIM inhibition when the classifier takes any level of inhibition as an input, as would be the case in real-world applications. The last two classifiers assess the classifiers’ ability to identify differences in each of the three groups. For each ROC curve, we calculated the area under the curve (AUC) statistic, which represents the probability that a randomly selected positive sample is ranked as more likely to have SIM inhibition than a randomly selected negative sample [28]. Although cPDR90 is also an area under the curve (of the PDRr curve), we will use AUC solely to refer to the area under the ROC curve.
(3) *Assessment of single and consensus classifiers.* We assessed the accuracy, sensitivity, specificity, and Matthew’s correlation coefficient (MCC) of each classifier at their optimal thresholds (the cutoff threshold that maximizes the sum of the sensitivity and specificity of the test [29]). The MCC is an alternative accuracy measurement that is preferred for unbalanced datasets and has a range of [−1,1] where 1 means perfect classification, 0 corresponds to a coin toss classifier, and −1 is perfect misclassification [30]. We further examined the accuracy, sensitivity, specificity, and Matthew’s correlation coefficient (MCC) of consensus classifiers consisting of each combination of the individual metrics $\rho $, cPDR90, *T*_50_ and ${T_{{\text{peak}}}}$ at their optimal thresholds. To generate these statistics for each participant in each experiment, we generated consensus diagnoses for each participant based on each combination of the individual classifiers. For example, assuming that a positive diagnosis of SIM inhibition is defined by *both* $\rho $ and cPDR90 ($\rho $ *∩*cPDR90) indicating inhibition or assuming that a positive diagnosis is defined by *either* $\rho $ and cPDR90 ($\rho $ $\mathop \cup \nolimits $ cPDR90) indicating inhibition. We assessed this for each possible combination of three classifiers at a time. For example, for *ρ*, cPDR90, and ${T_{50}}$ that is: *ρ* only, cPDR90 only, ${T_{50}}$ only, *ρ ∩* cPDR90, *ρ ∩* ${T_{50}}$, cPDR90 *∩* ${T_{50}}$, *ρ ∩* cPDR90 *∩* ${T_{50}}$, *ρ* $\mathop \cup \nolimits $ cPDR90, *ρ* $\mathop \cup \nolimits $ ${T_{50}}$, cPDR90 $\mathop \cup \nolimits $ ${T_{50}}$, and a majority rules classifier. For the majority rules classifier, a positive diagnosis was generated if at least two of the individual classifiers are positive. To compare consensus classifier performances for each of the three MLE doses, we generated this result for each of the same four comparison groups outlined in step 2. We repeated this for the 60, 90, and 120 min test lengths to assess classifier robustness to decreased data.

## 3. Results

### 3.1. Comparing model fits for 60, 90, 120, and 240 min tests

**Figure 1.**
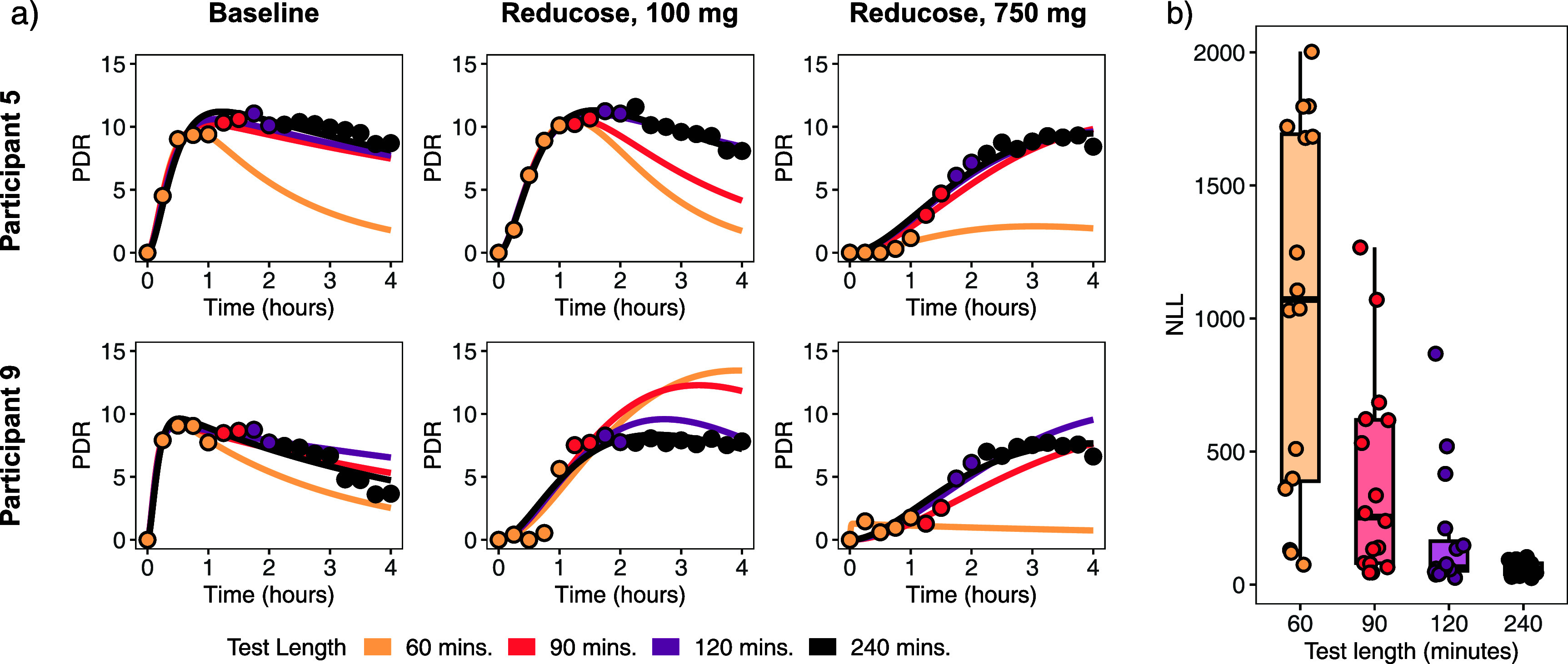
(a) Model best fits for 60, 90, 120, and 240 min test durations for two study participants (b) Boxplot of negative log-likelihoods (NLLs), a measure of how well the model fits the data, for each test duration, with larger values indicating poorer fit. Plots for all participants are given in figure S1 in the Supplementary material.

Projections from fitting the model only to the first 60 min of the data were consistently poor fits for the later data (illustrative examples given in figure 1(a), with full results in figure S1 in the Supplementary material). For the 60 min test duration, random variations present in each data point had a higher influence on the model fit than it did with longer test periods, causing model trajectories in hours 1–4 to be heavily impacted by these fluctuations. Additionally, the inability to observe the peak PDRr in the first hour—particularly for the 750 mg group—meant that *πρ* and *κ* were unidentifiable at this test duration, severely limiting the model’s inferential ability for later hours. While the 90 min test duration generally improved the fit somewhat, the improvement was not consistent across participants, and many curves fit to 90 min were poorly predictive of later dynamics. When comparing the NLLs between the models fit to data from each test length (figure 1(b)), we found substantial heterogeneity in the impact of test length on model fit, depending on the participant. The fits at shorter tests lengths were typically better in participants for whom the peak PDRr was reached within the respective test length (see figure S1 in the Supplementary material). In general, the projections from curve fit to the data from the first 120 min are very similar to the curves fit to the full data, with some outliers. In the following sections, we assessed how the improvement in model fit is reflected in the diagnostic capability of the test.

**Figure 2.**
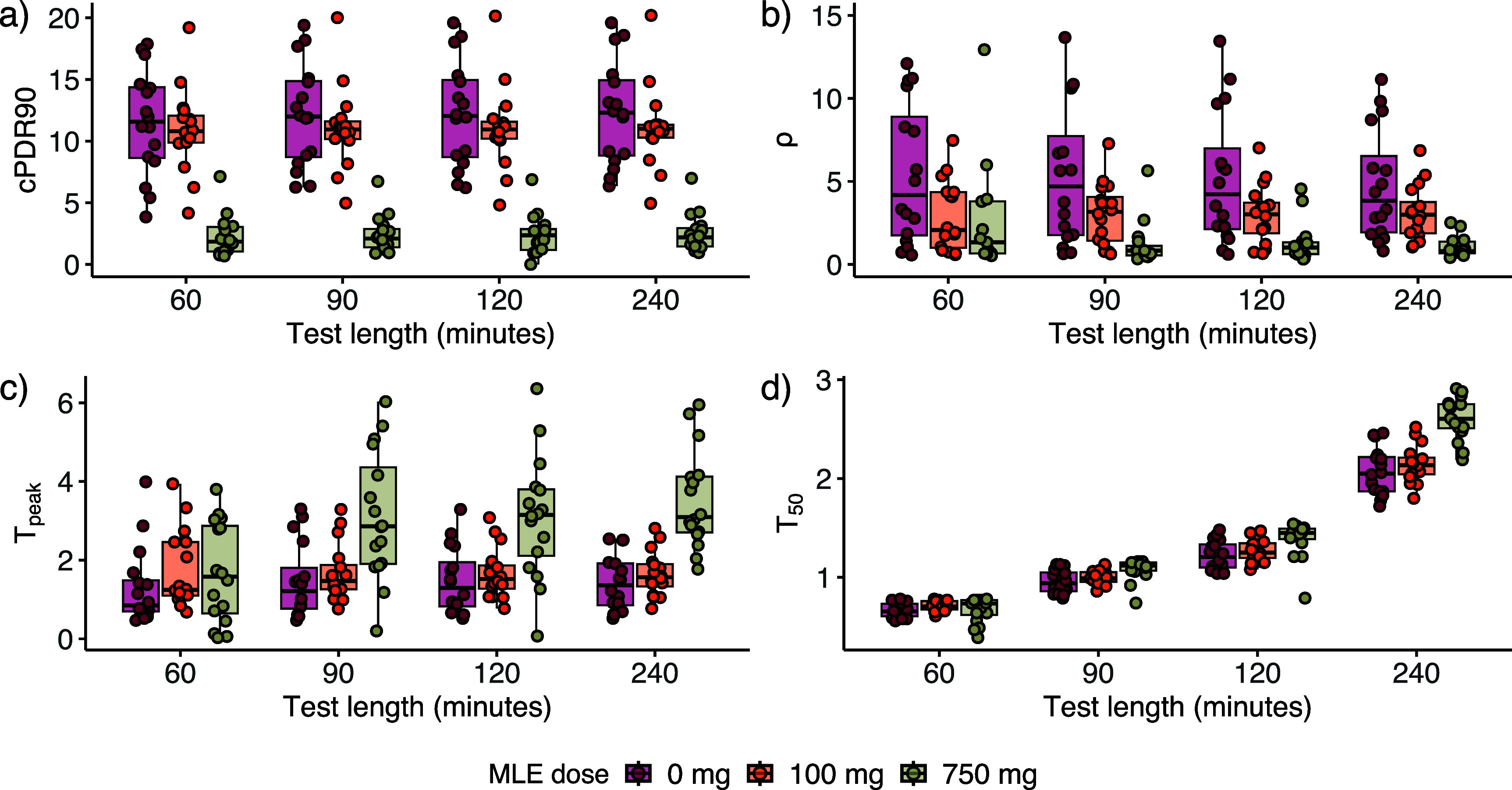
Classifier values for 60, 90, 120, and 240 min ^13^C-sucrose breath test durations for baseline, 100, and 750 mg doses of Reducose®, a mulberry leaf extract (MLE) that acts as a sucrase-isomaltase inhibitor for (a) cPDR90, (b) $\rho $, (c) ${T_{{\text{peak}}}}$, and (d) ${T_{50}}$.

We also plot the value of each classifier for each participant and test duration across the three MLE doses to visualize each classifier’s sensitivity to MLE dosage (figure 2). The plots for cPDR90 (figure 2(a)) show that this classifier has the strongest distinction between the lowest two doses (i.e. baseline or 100 mg MLE) and the 750 mg dose; however, the distinction between the baseline and 100 mg MLE dose is minor. By contrast, the figure for *ρ* (figure 2(b)) shows a better separation between the value of *ρ* and MLE dose, indicating that this classifier may be more sensitive to detecting lower MLE doses, which represent mild SIM inhibition.

### Comparing ROC curves for ρ, cPDR, time to 50% dose recovered (${T_{50}}$), and time to peak (${T_{{\text{peak}}}}$) for 60, 90, 120, and 240 min duration tests

3.2.

**Figure 3.**
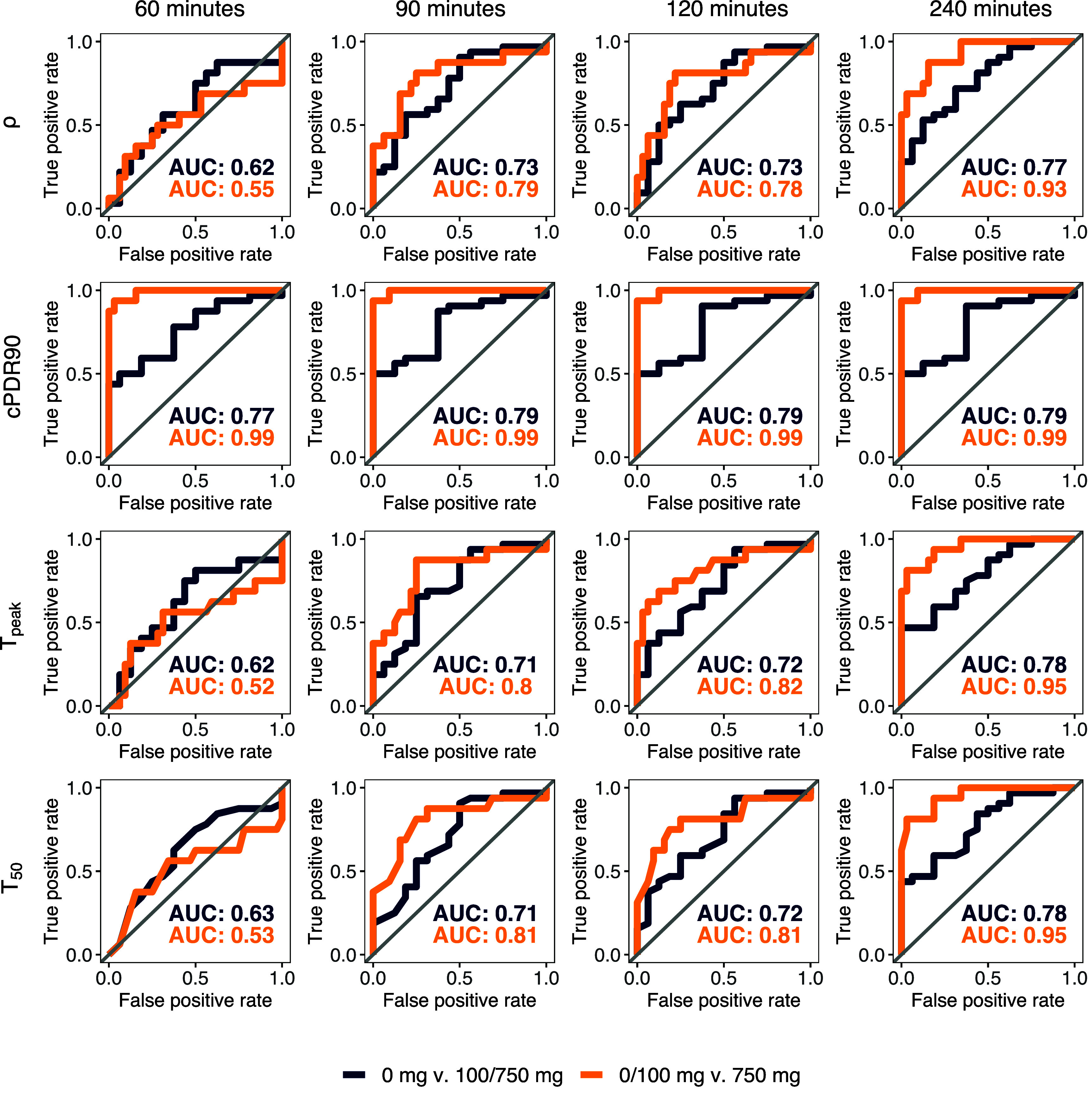
ROC curves for 60, 90, 120, and 240 min ^13^C-sucrose breath test durations for baseline versus either 100 or 750 mg doses of Reducose®, a mulberry leaf extract (MLE) that acts as a sucrase-isomaltase inhibitor (blue), and baseline or 100 mg MLE versus 750 mg MLE (orange).

We found that cPDR90 and cPDR60 outperformed cPDR120, and cPDR240 in the ROC curves (figure S2). Prior literature has used cPDR90, so, for consistency, we selected cPDR90 as the cPDR classifier to compare to *ρ,* ${T_{50}}$ and ${T_{{\text{peak}}}}$. Our ROC curves for baseline versus either 100 or 750 mg MLE (figure 3, blue) and baseline or 100 mg MLE versus 750 mg MLE (figure 3, yellow) showed that cPDR90 had the highest AUC for each test length and comparison group. The cPDR90 classifier also maintained the same AUC (0.99) for each test length for 0 or 100 mg v. 750 mg and only saw a slight decrease in the AUC for the other comparison group (0.79 at 240 min versus 0.77 at 60 min). The ROC curves corresponding to baseline versus 100 mg (figure S3) show that *ρ* outperforms cPDR90 for distinguishing mild SIM inhibition from none (AUC ranges: 0.61–0.66 for *ρ* and 0.55–0.60 for cPDR90). However, because *ρ* was not as accurate at distinguishing severe inhibition from no inhibition in these data (AUC range: 0.58–0.93), its AUC is always below the AUCs corresponding to cPDR90 in figure 3. Additional ROC curves assuming the data is available at 15 min for hours 0–1, every 30 min for hours 1–4 is available in the Supplementary material as an additional sensitivity analysis (figure S4).

### 3.3. Assessment of consensus classifiers

Table 1 shows the results of the consensus classifiers including cPDR90, *ρ*, and ${T_{50}}$, which were the three highest performing classifiers according to figure 2. The consensus classifiers including ${T_{{\text{peak}}}}$ are available in the SI appendix (tables S1–S4). Consistent with the results from the ROC curves, the performance statistics of the consensus classifiers (table 1) show that cPDR90 alone has the highest accuracy and MCC for each of the four MLE dose comparison groupings. However, for sensitivity, cPDR90 is outperformed by *ρ* and *T*_50_ for the baseline versus 100 mg group, and by *ρ* cPDR90 for 0 versus 750 mg and 0/100 versus 750 mg. For the shorter test durations, cPDR90 continues to be the best classifier for all comparison groups for the 120 min test length (table S1). However, *ρ* and ${T_{50}}$ surpass cPDR90 by the 90 and 60 min lengths for the baseline versus 100 mg and 0 v 100/750 mg comparison groups (tables S1 and S2). The consensus classifiers also perform better than the individual classifiers at these shorter test durations. For example, at the 60 min test duration, cPDR *∩* ${T_{{\text{peak}}}}$ and *ρ ∩* cPDR *∩* ${T_{{\text{peak}}}}$ had the highest accuracy and MCC for the baseline versus 100 mg group (table S1).

**Table 1.** Accuracy, sensitivity, specificity, and Matthew’s correlation coefficient (MCC) of consensus metrics for the 240 min duration test. The largest value(s) in each row is bolded.

	*ρ*	cPDR	${{\boldsymbol{T}}_{50}}$	*ρ ∩* cPDR	*ρ ∩* ${{\boldsymbol{T}}_{50}}$	cPDR *∩* ${{\boldsymbol{T}}_{50}}$	*ρ* $\mathop \cup \nolimits $ cPDR $\mathop \cup \nolimits $ ${{\boldsymbol{T}}_{50}}$	*ρ* $\mathop \cup \nolimits $ cPDR	*ρ* $\mathop \cup \nolimits $ ${{\boldsymbol{T}}_{50}}$	cPDR $\mathop \cup \nolimits $ ${{\boldsymbol{T}}_{50}}$	Majority rules
Accuracy											

0 v. 100 mg	0.66	**0.72**	0.66	**0.72**	0.66	**0.72**	0.66	0.66	0.66	0.66	0.66
0 v. 750 mg	0.88	**0.97**	0.91	0.91	0.91	0.91	0.94	0.94	0.88	**0.97**	0.91
0/100 v. 750 mg	0.85	**0.98**	0.92	0.94	0.92	0.94	0.90	0.9	0.85	0.96	0.92
0 v. 100/750 mg	0.71	**0.81**	0.62	0.73	0.62	0.62	0.79	0.79	0.71	**0.81**	0.73

Sensitivity											

0 v. 100 mg	**0.94**	0.81	**0.94**	0.81	**0.94**	0.81	**0.94**	**0.94**	**0.94**	**0.94**	**0.94**
0 v. 750 mg	0.88	0.94	0.81	0.81	0.81	0.81	**1.00**	**1.00**	0.88	0.94	0.81
0/100 v. 750 mg	0.88	0.94	0.81	0.81	0.81	0.81	**1.00**	**1.00**	0.88	0.94	0.81
0 v. 100/750 mg	0.72	**0.91**	0.44	0.72	0.44	0.44	**0.91**	**0.91**	0.72	**0.91**	0.72

Specificity											

0 v. 100 mg	0.38	**0.63**	0.38	**0.63**	0.38	0.62	0.38	0.38	0.38	0.38	0.38
0 v. 750 mg	0.88	**1.00**	**1.00**	**1.00**	**1.00**	**1.00**	0.88	0.88	0.88	**1.00**	**1.00**
0/100 v. 750 mg	0.84	**1.00**	0.97	**1.00**	0.97	**1.00**	0.84	0.84	0.84	0.97	0.97
0 v. 100/750 mg	0.69	0.63	**1.00**	0.75	**1.00**	**1.00**	0.56	0.56	0.69	0.62	0.75

MCCs											

0 v. 100 mg	0.38	**0.45**	0.38	**0.45**	0.38	**0.45**	0.38	0.38	0.38	0.38	0.38
0 v. 750 mg	0.75	**0.94**	0.83	0.83	0.83	0.83	0.88	0.88	0.75	**0.94**	0.83
0/100 v. 750 mg	0.69	**0.95**	0.81	0.86	0.81	0.86	0.80	0.80	0.69	0.91	0.81
0 v. 100/750 mg	0.39	**0.56**	0.45	0.45	0.45	0.45	0.51	0.51	0.39	**0.56**	0.45

## 4. Discussion

In this analysis, we leveraged a mechanistic model to compare the performance of traditional, empirical classifiers (i.e. cPDR90, ${T_{50}}$ and ${T_{{\text{peak}}}}$) of ^13^C-SBT breath test to that of a mechanistic, pharmacokinetic model-based classifier. We found that, under typical data variation, 60 min duration tests were insufficient to adequately project breath trajectories, primarily due to limited ability to observe some of the post-peak PDRr trajectory in these time lengths (figure 1). Thus, we recommend ^13^C-SBT future protocols use a 120 min or longer test duration. For the ^13^C-SBT, test durations up to 240 min saw enhanced accuracy and improvement in the performance of the ${T_{50}},\,{T_{\text{peak}}}$ and model-based classifier, but the ability to estimate SIM activity from a shorter-duration test supports the wider use of the ^13^C-SBT for gut dysfunction research and, potentially, for future clinical usage. However, other ^13^C breath tests may have different recommended durations if the distribution of peak PDRr is different for a different isotopic tracer, so further study of potential tracers could identify a substrate with a further reduced testing burden.

Our results from the classifier performance comparison show that cPDR90 was the best classifier (by AUC) at each test length, compared to *ρ,* ${T_{50}},$ and ${T_{{\text{peak}}}}$ (figure 2). These results suggest that, even though cPDR is not directly measuring the underlying biological mechanisms, slow cumulative recovery of the breath is highly informative. We also found that the consensus classifiers generally performed worse than the individual ones, largely because cPDR90 was highly accurate on its own for this population. However, as we see in equation (2), the cumulative percent dose recovery is highly dependent on *κ,* the fraction of tracer that is excreted through the breath. Hence, the performance of cPDR will be highly sensitive to variations in this fraction or, as we previously showed [23], to potential misestimation of the production rate of CO_2_, V_CO2_, which is estimated based on body size [26]. As a result, associations between cPDR and demographic or anthropometric variables may be introduced through differential bias in VCO_2_ estimates. This potential association may limit the applicability of the cPDR as a classifier of the ^13^C-SBT as a test of EED in young children, because poorer growth is posited to be a key consequence of EED. (We will explore anthropometric and demographic associations with breath curve dynamics in future work). Hence, we caution against taking our results as evidence that cPDR90 is the only classifier needed. Although it performed the best in this healthy, adult population with artificially induced SIM inhibition, it is not certain that it is the best classifier in children with gut dysfunction. Additionally, we note that both *ρ* and ${T_{{\text{peak}}}}$ outperform cPDR90 for model sensitivity (table 1) and for distinguishing the 100 mg dose from baseline (figure S2). Currently, it is unknown whether SIM inhibition in typical a case of EED or other gut dysfunction is more similar to the inhibition induced by the 100 mg MLE dose or the 750 mg dose.

We found that some classifiers were quite accurate at shorter test lengths or even had a higher AUC at shorter test lengths. For example, the ${T_{{\text{peak}}}}$ AUC for 0 mg v. 100 mg has a higher AUC (0.68) when generated from the 60-minute data as opposed to the 240 min data (AUC = 0.61). However, this result does not necessarily indicate that those classifiers were robust to a shorter test length. Rather, this behavior is a data artifact: the curves estimated at the shorter test lengths are often poor fits to the full breath curve (figure S1), and thus they happen to have better classifier performance only by accident. The same classifier might perform drastically worse on a different dataset for that test duration. This phenomenon is not a limitation of our analysis but a limitation of short-duration breath tests, and it has implications for future studies. Participants do not always complete the full breath collection protocol, but researchers may want to include the data that were collected. We advise having a clear exclusion criterion in ^13^C-SBT studies for participants who do not complete at least 90 min of breath collection.

The primary strength of this study is the crossover study design. The experimental design artificially induced SIM inhibition in the study participants, making the comparison between experiments unconfounded by other factors that would be likely present in cases and controls from separate populations. However, because the data is from healthy adult participants for whom SIM was experimentally inhibited, the performance of the classifiers may be different from the target population, i.e. children in low-resource settings, which means that the external generalizability may be limited. In addition, the small samples size makes the results more sensitive to random measurement error. For the ^13^C-SBT to move from being a specialized research tool to wider usability, further research that includes a larger sample size and inclusion of study participants from the target population will be needed. Our results facilitate this work by suggesting a shortened, 120 min test duration, that may be more feasible for infants and young children compared to the prior, standard 4 h test.

## 5. Conclusion

We assessed the performance of three empirical classifiers, cPDR90, ${T_{50}}$, and *T*_peak_, and one model-based classifier, *ρ* for the ^13^C-SBT over different test lengths. Based on curves fit to different test lengths, we recommend that ^13^C-SBT protocols include 120 min or longer test durations and that participants who collect less than 90 min of breath be excluded. We found that, overall, cPDR90 was the most accurate classifier in these data; however, limitations of this classifier include uncertainty around its performance in the target population and lower sensitivity in detecting cases of mild SIM inhibition. The model-based classifier $\rho $ addresses both concerns because it is more reflective of the underlying biological processes giving rise to the PDRr curves. We recommend multiple classifiers continue to be considered in future work assessing the performance of the ^13^C-SBT as a diagnostic test of EED or other dysfunctions that reduce SIM activity.

## Data Availability

The data that support the findings of this study are openly available at the following URL/DOI: https://doi.org/10.5281/zenodo.8387995.
